# Supplementary material for: The Interplay between Bluetongue Virus Infections and Adaptive Immunity
Source: Viruses. 2021 Jul 31;13(8):1511. doi: 10.3390/v13081511 (PMC8402766; doi:10.3390/v13081511)
Supplement: Supplementary file 1 [file viruses-13-01511-s001.zip › Supplemental File S1.pdf]

Supplementary File S1

Material and methods

Virus and sheep experimental infection

Bluetongue virus (BTV) serotype 8 (Belgium/06) was used in all experiments. Virus stock growth, titration and inactivation are detailed elsewhere [1–3]. Briefly, BTV-8 stocks were obtained by infection of BHK-21 cells (ATCC) at multiplicity of infection (MOI) 0.1. When cytopathic effect was observed (typically at 48h), culture supernatants and cellular fraction were harvested frozen/thawed 3 times and clarified by centrifugation at 2000xg, for 10min. Virus stocks were tittered by standard plaque assays in Vero cells in semi-solid medium. BTV-8 stock was inactivated with 3mM binary ethyleneimine.

All the animal experiments were carried out in a disease-secure isolation facility (BSL3) at the *Centro de Investigación en Sanidad Animal* (CISA), in strict accordance with the recommendations in the guidelines of the Code for Methods and Welfare Considerations in Behavioural Research with Animals (Directive 86/609EC; RD1201/2005) and all efforts were made to minimize suffering. Experiments were approved by the Committee on the Ethics of Animal Experiments (CEEA) (Permit number: 10/142792.9/12) of the Spanish *Instituto Nacional de Investigación y Tecnología Agraría y Alimentar*ia (INIA) and the "*Comisión de ética estatal de bienestar animal*" (Permit numbers: CBS2012/06 and PROEX 228/14).

Twelve 18-month old female Churra breed sheep obtained from a certified provider were housed in the Animal Facility at CISA-INIA, and randomly divided into 2 groups. One group (BTV-8; *n=8*) received 2×10^6 plaque forming units of BTV-8 intradermally at several sites, while the second group (Mock; *n*=*4*) received similar intradermal mock-injections with PBS. Animals were thereafter

examined daily for clinical signs and daily rectal temperature recorded. For the duration of the experiment a clinical score was assigned to each animal as described in [3]. At the end of the experiment, sheep were euthanized by intravenous administration of T61 (4–6 mL/50Kg bw) following intramuscular xylazona (0.3 mg/Kg bw) to minimize suffering.

RNA extraction and quantitative PCR

Total RNA was extracted from blood samples using Trizol Reagent Solution (Invitrogen) following the manufacturer´s protocol. Reverse transcription quantitative PCR (RT-qPCR) was performed using Luna® Universal One-Step RT-qPCR Kit (New England Biolabs). Primers Forward 5`-GTTGAATTGGCAAAGGAGGCAATG-´3 and Reverse 5´-GGGATGATGGATGAGGCCGTG-3´ were used to amplify a 165pb fragment of the segment 5 of BTV, which encodes the non-structural protein 1 (NS1). Quantifications were performed on a MX3005P thermocycler (Agilent).

PBMC isolation and ovine IFN-γ ELISPOT assays

PBMC isolation was performed by standard centrifugation method over Ficoll cushion as described in [3]. Ovine IFN-γ ELISPOT assays were performed as described in [2]. Briefly, MSIPS4510 plates (Millipore) were coated with 5 μg/mL anti-bovine IFN-γ antibody (MT17.1, Mabtech, Sweden). Sheep PBMC were plated at a density of 2×10^5 cells per well and incubated with inactivated BTV (equivalent to MOI 1 prior to inactivation) or medium as negative control. Plates were incubated with biotin-labelled anti-ovine IFN-γ antibody (MT307-biotin, Mabtech, Sweden) and developed with streptavidin conjugated to alkaline phosphatase (ExtrAvidin-AP, Sigma, USA). Plate membranes were revealed using SigmaFAST BCIP/NBT (Sigma).*Flow cytometry*

$CD4^+$, $CD8^+$, B-cell$^+$, $CD21^+$, $WC1^+$ (γδ T-cells) and $CD335^+$ $CD3^-$ (NK cells) populations were stained with primary antibodies diluted in PBS stain buffer

(PBS + 2% FBS + 0.03% sodium azide) for 20 min on ice, followed by 1% paraformaldehyde (PFA) fixation. The following antibodies were used for this purpose: anti-CD4 (clone 44.38, Biorad), anti-CD8 (clone 38.65, Biorad), anti-Bcell (BAQ44A, Kingfisher Biotech), anti-CD21 (LB21, Biorad), anti-WC1 (19.19, Biorad), anti-CD335 (EC1.1, Biorad) and anti-CD3 (CD3-12, Biorad). All appropriate isotype controls were included. Samples were acquired on a FACScalibur flow cytometer and data was analyzed with FlowJo v10 software (FlowJo, LLC).

Anti-VP7 IgG ELISA

Total anti-VP7 IgG quantification was performed as described by Martin et al. (Martin et al., 2015). Briefly, MaxiSorp plates (Thermofisher) were coated with 200ng/well of baculovirus-expressed BTV-8 VP7 protein in carbonate buffer pH 9.6, O/N, 4ºC. Plates were blocked with PBS + 0.05% Tween (PBS-T) + 5% milk for 1 h, RT, washed with PBS-T and incubated with serial dilutions (1:100 to 1:1600) of D7pc and D22pc sheep sera in PBS-T + 2% milk for 2 h, RT. Pre-immune sera from each animal (1:100 dilution) were used as base signal controls. Appropriate negative and positive controls were added. After that, plates were washed and incubated with donkey anti-sheep IgG-HRP (Biorad) in PBS-T + 2% milk for 1 h, followed by signal developement with 3, 3′, 5, 5′-Tetramethyl-benzidine (TMB) (Thermofisher). After 15 min, reaction was stopped with H_2SO_4 3M. Absorbance was read at 450nm in a FLUOstar Omega microplate reader. Total anti-VP7 antibodies were expressed as the lowest dilution rendering an absorbance value above the cut-off level, established for each animal as twice the value of pre-immune sera.

References

1. Rojas, J.M.; Rodriguez-Calvo, T.; Pena, L.; Sevilla, N. T cell responses to bluetongue virus are directed against multiple and identical CD4+ and CD8+ T cell epitopes from the VP7 core protein in mouse and sheep. *Vaccine* **2011**, *29*, 6848–6857.

2. Rojas, J.M.; Pena, L.; Martin, V.; Sevilla, N. Ovine and murine T cell epitopes from the non-structural protein 1 (NS1) of bluetongue virus serotype 8 (BTV-8) are shared among viral serotypes. *Vet. Res.* **2014**, *45*, 30.
3. Martin, V.; Pascual, E.; Avia, M.; Pena, L.; Valcarcel, F.; Sevilla, N. Protective Efficacy in Sheep of Adenovirus-Vectored Vaccines against Bluetongue Virus Is Associated with Specific T Cell Responses. *PLoS ONE* **2015**, *10*, e0143273
