# Supplementary figures and images for: The Interplay between Bluetongue Virus Infections and Adaptive Immunity
Source: Viruses. 2021 Jul 31;13(8):1511. doi: 10.3390/v13081511 (PMC8402766; doi:10.3390/v13081511)

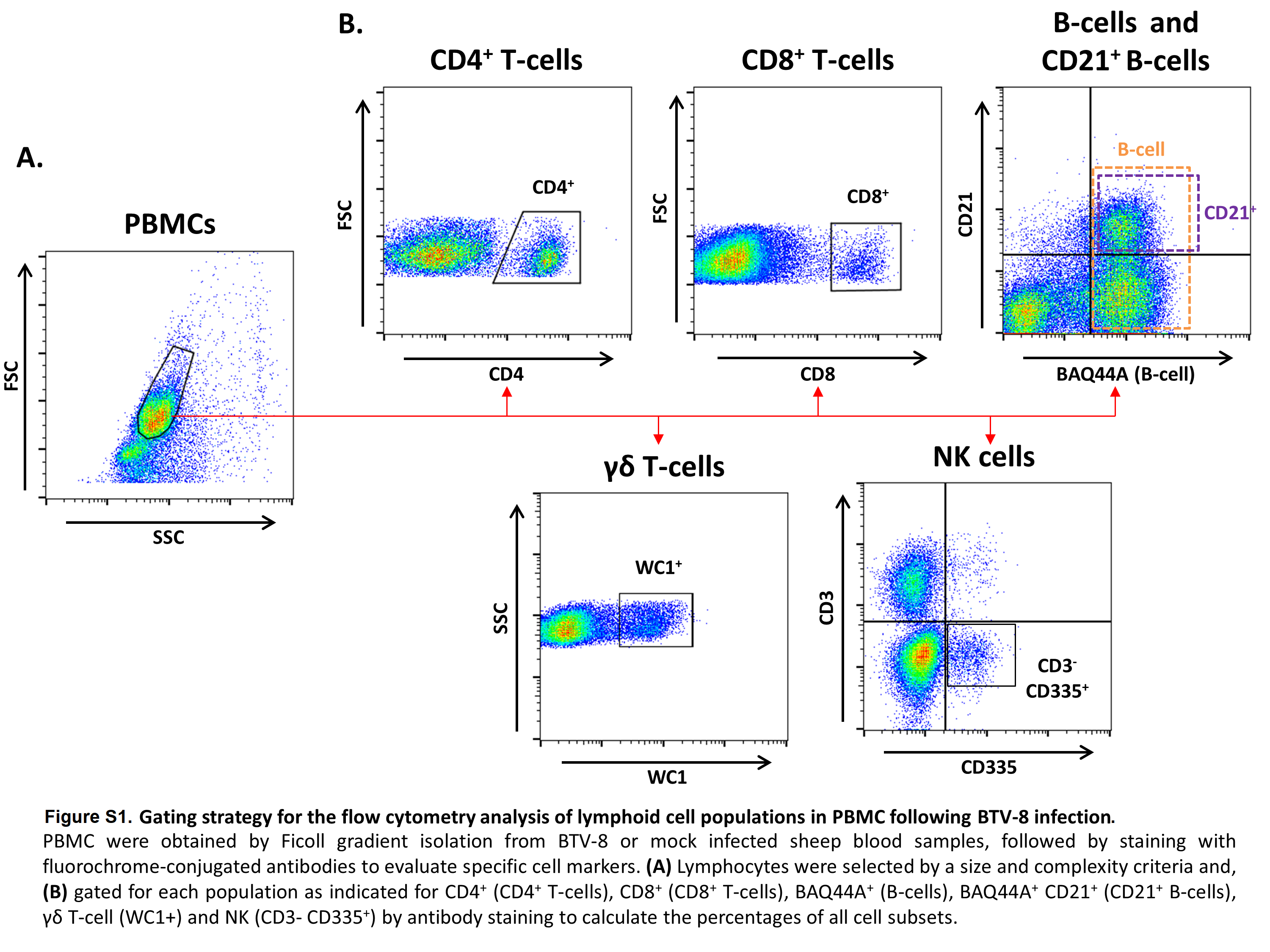

Supplement: Supplementary file 1 [file viruses-13-01511-s001.zip › Figure S1.png]
